# *Juniperus communis* L. Needle Extract Modulates Oxidative and Inflammatory Pathways in an Experimental Model of Acute Inflammation

**DOI:** 10.3390/molecules31020247

**Published:** 2026-01-11

**Authors:** Dinu Bolunduț, Alina Elena Pârvu, Andra Diana Cecan, Anca Elena But, Florica Ranga, Marcel Pârvu, Iulia Ioana Morar, Ciprian Ovidiu Dalai

**Affiliations:** 1 Department of Morphofunctional Sciences, Faculty of Medicine, “Iuliu Hațieganu” University of Medicine and Pharmacy, 400012 Cluj-Napoca, Romania; bolundut_dinu@elearn.umfcluj.ro (D.B.); parvualinaelena@umfcluj.ro (A.E.P.); ancabut@umfcluj.ro (A.E.B.); iulia.morar@umfcluj.ro (I.I.M.); 2 Department of Medical Oncology, “Ion Chiricuță” Institute of Oncology, 34-36 Republicii Street, 400015 Cluj-Napoca, Romania; 3 Department of Food Science, University of Agricultural Sciences and Veterinary Medicine of Cluj-Napoca, Calea Mănăștur 3-5, 400372 Cluj-Napoca, Romania; florica.ranga@usamvcluj.ro; 4 Department of Biology, Babeș-Bolyai University, 400015 Cluj-Napoca, Romania; marcel.parvu@ubbcluj.ro; 5 Department of Surgical Sciences, Faculty of Medicine and Pharmacy, University of Oradea, 410087 Oradea, Romania; cipridalai@gmail.com

**Keywords:** phytochemicals, inflammation, antioxidants, oxidative stress, cytokines

## Abstract

*Juniperus communis* L. is a conifer widely used in traditional European medicine for the management of inflammatory disorders. However, its effects on oxidative stress and inflammation remain incompletely characterized. The present study investigated the antioxidant and anti-inflammatory potential of an ethanolic needle extract of *J. communis* using in vitro assays and an in vivo model of acute inflammation induced by turpentine oil in rats. Phytochemical profiling by HPLC–DAD–ESI–MS revealed a polyphenol-rich extract dominated by flavonols, flavanols, and hydroxybenzoic acids, with quercetin derivatives and taxifolin as major constituents. In vitro analyses demonstrated radical-scavenging and reducing capacities, exceeding or comparable to reference antioxidants in DPPH, hydrogen peroxide, ferric-reducing, and nitric oxide scavenging assays. In vivo, both therapeutic and prophylactic administration of the extract significantly attenuated oxidative and nitrosative stress, as evidenced by reductions in total oxidant status, oxidative stress index, malondialdehyde, advanced oxidation protein products, nitric oxide, 3-nitrotyrosine, and 8-hydroxy-2′-deoxyguanosine, alongside restoration of total antioxidant capacity and thiol levels. These effects were concentration-dependent. Concomitantly, inflammatory signaling was suppressed, with decreased NF-κB activity and reduced levels of interleukin-1β and interleukin-18. These results support the use of these extracts, whose benefits have been observed in traditional medicine, providing scientific support for the anti-inflammatory and antioxidant capacity of *J. communis* extract.

## 1. Introduction

For centuries, European traditional medicine has used conifer extracts as some of its most valuable therapeutic agents. Communities in the Alpine and Balkan regions, for example, applied the balsams and resins of *Picea abies*, *Larix decidua*, and *Pinus nigra* to treat wounds, ulcers, and rheumatic pain [1]. *Juniperus communis* (common juniper) has a rich ethnomedicinal history. Its berries and needle infusions have been widely used as diuretics and antiseptics and to relieve inflammatory conditions such as arthritis, gout, and migraines [2]. The anti-inflammatory potential of *J. communis* has been well recognized in European folk medicine, and *J. communis* preparations have long been used to manage respiratory and rheumatic conditions. These historical observations are increasingly supported by modern research, which confirms the antioxidant, antiseptic, analgesic, and decongestant effects of *J. communis* in various experimental models [3].

The academic community worldwide has shown increasing interest in the therapeutic properties of conifers, and they have been the subject of many in vitro tests that have provided evidence that justifies the observed in vivo effects. Researchers have begun to identify the bioactive constituents of *J. communis*, which is rich in aromatic oils and phenolic compounds, including monoterpenes (such as α-pinene, sabinene, β-myrcene), flavonoids and hydroxycinnamic acids, known for their antioxidant and anti-inflammatory properties [4]. In particular, an isolated biflavonoid (amentoflavone) from *J. communis* has demonstrated antiarthritic and anti-inflammatory activity in vivo, partly by modulating key inflammatory pathways [5]. *J. communis* essential oil, dominated by terpenes, can inhibit pro-inflammatory NF-κB signaling and neutrophil activation [6]. In line with its traditional uses, extracts of *J. communis* have demonstrated significant anti-inflammatory effects in animal studies; for example, *J. communis* extracts reduced carrageenan-induced paw edema in rats and suppressed the release of cytokines such as IL-1β, IL-6, and TNF-α in a dextran/kaolin-induced inflammation model [7]. The in vitro and in vivo findings provide scientific support for the ethnobotanical applications of *J. communis*, reinforcing the rationale for further exploration of its therapeutic potential.

Inflammation and oxidative stress are two pathophysiological processes that represent the cornerstone of numerous acute and chronic diseases. The two processes are closely interconnected. An inflammatory response is essential for host defense. Activated neutrophils and macrophages generate reactive oxygen and nitrogen species (ROS/RNS) as part of the response. When released in excess, ROS/RNS can cause oxidative stress and tissue damage by oxidizing lipids, proteins, and DNA [8]. Oxidative tissue injury produces secondary signals and markers of damage; for example, 3-nitrotyrosine (3-NT), a stable end product of peroxynitrite-mediated tyrosine nitration, accumulates when RNS are produced in excess. Furthermore, oxidative stress will amplify inflammation by activating redox-sensitive signaling pathways, like nuclear factor kappa B (NF-κB), one of the key transcription factors in immunity. Once activated, NF-κB upregulates pro-inflammatory cytokines and enzymes (e.g., IL-1, TNF-α, COX-2), thereby intensifying the inflammatory response [9]. These oxidative modifications can further stimulate inflammatory pathways (e.g., through recognition of patterns of damaged molecules), creating a vicious cycle in which inflammation generates oxidative stress, and oxidative stress propagates inflammation. Therefore, breaking this self-perpetuating cycle through intervention with agents that possess both antioxidant and anti-inflammatory properties is a key therapeutic strategy in acute inflammation [10].

A number of biomolecular markers can be measured to assess the degree of oxidative stress and inflammation during acute inflammation, many of which also represent potential therapeutic targets. To obtain an integrative measure of the overall redox state, the total oxidant status (TOS) and total antioxidant capacity (TAC) can be quantified. TOS and TAC, respectively, reflect the cumulative levels of all oxidants and all antioxidants in a sample, and their ratio, the oxidative stress index (OSI), provides a balanced indicator of oxidative burden versus antioxidant defenses. An elevated OSI (i.e., high TOS alongside low TAC) is characteristic of acute inflammatory states where oxidative stress predominates [11]. 8-Hydroxy-2′-deoxyguanosine (8-OHdG) reflects oxidative DNA damage due to ROS-mediated guanine oxidation, and its increased levels serve as an indicator of DNA injury in inflamed tissues [12]. During acute inflammation, overproduction of nitric oxide (NO) by inducible NO synthase (iNOS) in immune cells contributes to nitrosative stress. NO readily reacts with superoxide anion to form peroxynitrite (ONOO^−^), a potent oxidant that nitrates tyrosine (yielding 3-NT) and further damages tissues [13]. 3-Nitrotyrosine (3-NT) is a reliable biomarker of nitrosative stress formed by the reaction of peroxynitrite with tyrosine residues on proteins; elevated 3-NT indicates excessive RNS production and correlates with inflammation-induced protein dysfunction [14]. Malondialdehyde (MDA), a reactive dialdehyde byproduct of polyunsaturated lipid peroxidation, is another classic marker of oxidative stress. High MDA levels reflect ROS-induced membrane lipid damage and correlate with the intensity of inflammation and tissue injury. Advanced oxidation protein products (AOPPs), oxidatively modified albumin and plasma proteins are markers of protein oxidation that also act as pro-inflammatory signals [15]. AOPPs can trigger an oxidative burst and cytokine release from neutrophils and monocytes, thus perpetuating inflammation if not cleared [16].

NF-κB, as mentioned, is a redox-sensitive transcription factor that orchestrates the expression of many pro-inflammatory mediators; persistent NF-κB activation under oxidative conditions leads to sustained cytokine release and has made NF-κB a central target for anti-inflammatory interventions [17]. The pro-inflammatory cytokines interleukin-1β (IL-1β) and interleukin-18 (IL-18) are produced via activation of the NLRP3 inflammasome by NF-κB, a process triggered in part by ROS and danger-associated molecular patterns (DAMPs) or pathogen-associated molecular patterns (PAMPs). Upon assembly of the NLRP3 inflammasome, caspase-1 is activated to cleave pro-IL-1β and pro-IL-18 into their active forms, which then amplify the inflammatory cascade. In effective anti-inflammatory treatments, reductions in IL-1β and IL-18 levels are often observed, underscoring their role as mediators of acute inflammation [18].

By monitoring these biomarkers, the extent of oxidative damage and the magnitude of the inflammatory response during an acute inflammatory episode can be assessed. An acute inflammatory process that is not properly treated may progress to a chronic state [19]. Chronic inflammation is characterized by persistent overproduction of ROS/RNS and continuous activation of inflammatory pathways, leading to ongoing molecular damage, fibrotic tissue changes, or even carcinogenesis over time. Therefore, the persistence of the inflammatory process is implicated in all stages of tumor development, and an inflammatory microenvironment is considered a hallmark of cancer [20]. Consequently, early modulation of the oxidative inflammatory cycle with phytochemicals that possess antioxidant and anti-inflammatory activities is a promising approach, not only for controlling acute inflammatory episodes but also for preventing their escalation into chronic, degenerative conditions [21].

Considering the traditional use of the species *J. communis* and the preliminary evidence supporting its therapeutic potential, the present study had the following objectives. First, the needle extract of *J. communis* was phytochemically characterized, and then its in vitro antioxidant activity was evaluated. Subsequently, the in vivo evaluation of the extract’s antioxidant and anti-inflammatory potential is carried out in a model of acute inflammation induced by turpentine oil in the rat, both for therapeutic and prophylactic purposes. This dual experimental approach (prophylactic and therapeutic) will elucidate whether the *J. communis* needle extract can attenuate or prevent oxidative stress and inflammation, thereby scientifically validating its traditional medicinal use.

## 2. Results

### 2.1. Phytochemical Analysis

The total flavonoid content (TFC) and total phenolic content (TPC) of the *Juniperus communis* needle extract were quantified spectrophotometrically. The extract exhibited a high flavonoid content, with a TFC value of 145.38 ± 28.28 mg quercetin equivalents (QE)/g dry weight, indicating a substantial presence of flavonoid compounds. In contrast, the total phenolic content was lower, reaching 1.75 ± 0.18 mg gallic acid equivalents (GAE)/g dry weight.

**Figure 1:**
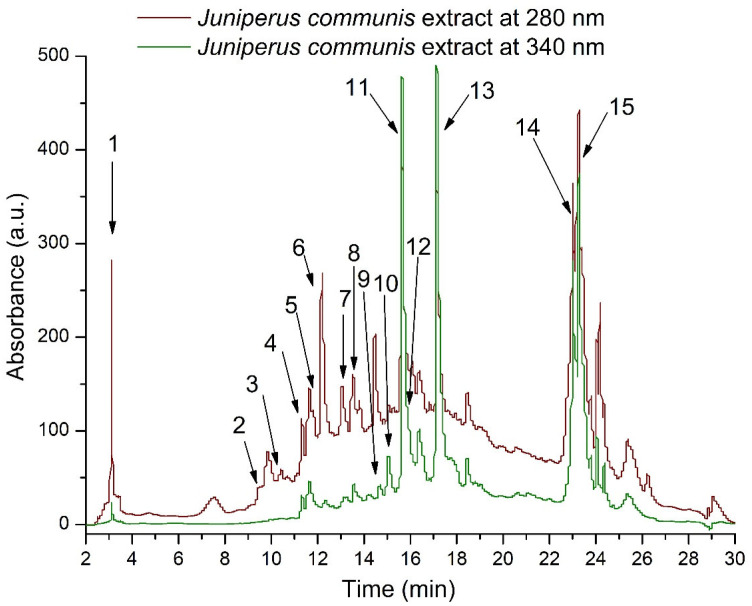
Representative HPLC–DAD chromatogram of *J. communis* extract at 280 and 340 nm. Peak identification is presented in Table 1.

Figure 1 represents the HPLC chromatogram of *J. communis* extract recorded at 280 nm and 340 nm. In total, 23 compounds were annotated, predominantly flavanols and flavonols, alongside hydroxybenzoic acids. Among the quantified compounds, flavonols accounted for the largest proportion, comprising approximately 66% of the total phenolic content. Flavanols represented an additional 30% of the phenolics detected by HPLC, whereas hydroxybenzoic acids constituted 4% of the total identified phenolic compounds.

Within the major identified flavonol class, quercetin derivatives represented 23% of total phenolic compounds, while the dihydroflavonol taxifolin (or dihydroquercetin) accounted for another 14%. Further, myricetin derivatives were almost in the same percentage as kaempferol derivatives, accounting for approximately 14 and 12% of the quantified phenolics (Table 1). Isohamentin derivatives were poorly represented (approximately 2% of total phenolics). Next, flavanol dimers were present at moderate levels, accounting for 156.58 μg/mL (approximately 11% of total phenolics), while monomeric flavan-3-ols were detected in slightly higher amounts, on average 260.66 μg/mL (approximately 18.95% of total phenolic compounds).

### 2.2. In Vitro Antioxidant Capacity Testing

*J. communis* extract exhibited significant antioxidant and radical-scavenging activity across assays (Table 2). Relative to Trolox, DPPH radical-scavenging, H_2_O_2_ scavenging, and ferric-reducing power were higher (*p* < 0.01, *p* < 0.01, and *p* = 0.030, respectively). Compared with quercetin, NO scavenging was markedly higher (*p* < 0.01).

### 2.3. In Vivo Antioxidant and Anti-Inflammatory Activity

**Figure 2:**
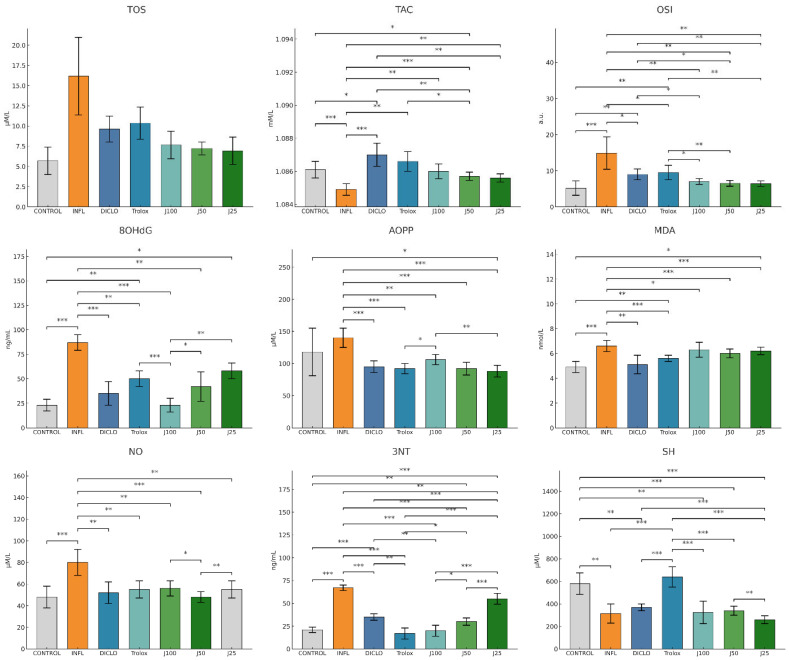
The therapeutic effect of *J. communis* extracts on oxidative stress parameters. * *p* < 0.05; ** *p* < 0.01; *** *p* < 0.001; TOS—Total oxidative status; TAC—Total antioxidant capacity; OSI—Oxidative stress index; MDA—Malondialdehyde; AOPP—Advanced oxidation protein products; 8OHdG—8-hydroxy-2′-deoxyguanosine; NO—Nitric oxide; 3NT—3-nitrotyrosine; SH—Total thiols; DICLO—Diclofenac (10 mg/kg); Trolox—Trolox (50 mg/kg); J100*—J. communis* extract 100%; J50—*J. communis* extract 50%; J25—*J. communis* extract 25%; INFL—Inflammation induced by turpentine oil.

Acute inflammation was induced with intramuscular administration of turpentine oil. As shown in Figure 2, turpentine oil produced oxidative imbalance versus the control group, with significant elevations of TOS, OSI, MDA, NO, 3-NT, 8-OHdG, and AOPP, accompanied by reductions in TAC and SH compared to control group.

Therapeutic administration of the *J. communis* needle extract attenuated this imbalance in a concentration-dependent manner. *J. communis* extract 100% (J100) yielded the largest improvements, lowering lipid and protein oxidation, nitrosative and DNA damage (MDA, AOPP, NO, 3-NT, 8-OHdG, OSI, TOS) while restoring antioxidant status (TAC, SH) toward INFL. *J. communis* extract 50% (J50) produced consistent but more modest corrections, whereas *J. communis* extract 25% (J25) achieved only partial recovery. Diclofenac and Trolox confirmed reference efficacy, with effects overlapping those of J100 for several endpoints.

**Figure 3:**
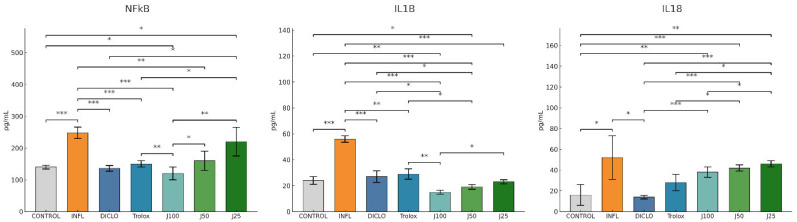
The therapeutic effect of *J. communis* extracts on inflammatory marker parameters. * *p* < 0.05; ** *p* < 0.01; *** *p* < 0.001; NF-κB—Nuclear factor kappa B; IL-18—Interleukin 18; IL-1B—Interleukin 1 beta; DICLO—Diclofenac (10 mg/kg); Trolox—Trolox (50 mg/kg); J100—*J. communis* extract 100%; J50*—J. communis* extract 50%; J25—*J. communis* extract 25%; INFL—Inflammation induced by turpentine oil.

Consistent with the biochemical findings, Figure 3 shows that inflammatory mediators NF-κB, IL-1β, and IL-18 were markedly increased in the INFL group versus. Therapeutic treatment with *J. communis* suppressed these cytokines in a concentration-dependent manner. J100 produced the strongest inhibition vs. INFL, approaching the levels achieved by diclofenac and Trolox; J50 remained significantly effective (*p*  <  0.05), while J25 showed a limited, though noticeable, reduction. The therapeutic regimen with *J. communis* ameliorated oxidative stress and dampened inflammation, with efficacy increasing with concentration.

**Figure 4:**
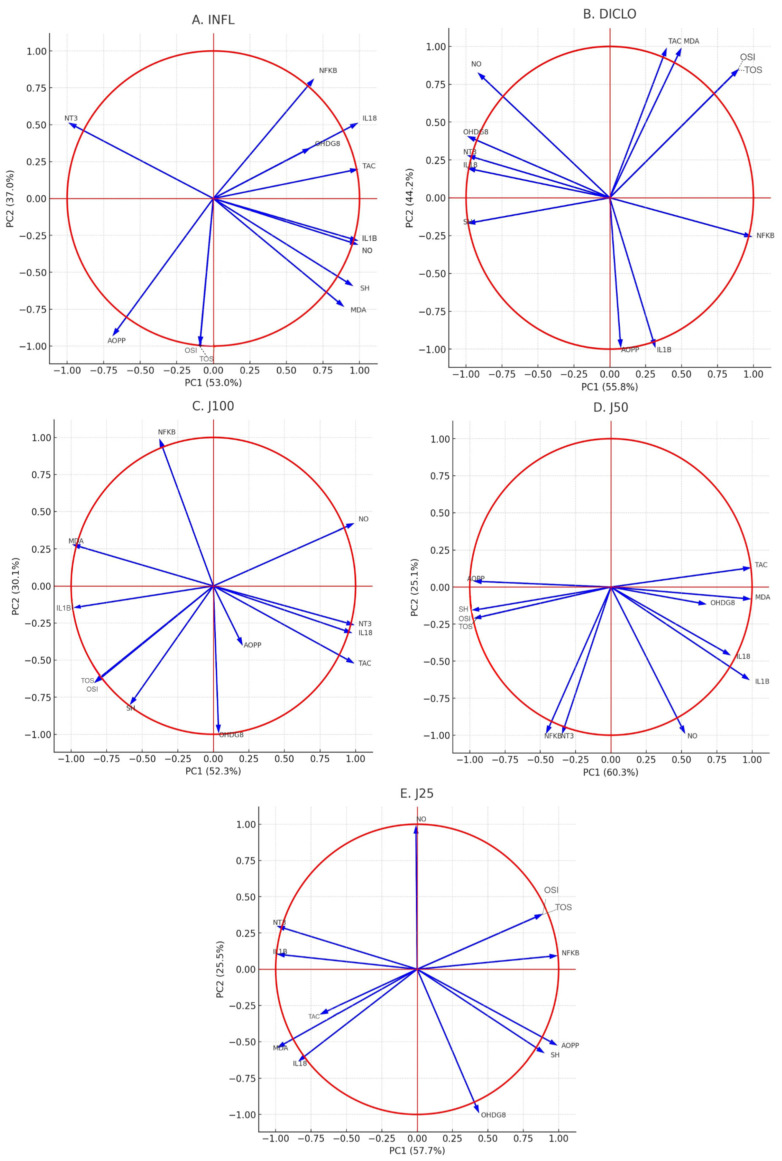
PCA correlation circles of oxidative stress and inflammatory markers in experimental groups. Vectors represent the contribution and correlation of each biomarker with the first two principal components (PC1 and PC2). (**A**) INFL group; (**B**) DICLO group; (**C**) J100% group; (**D**) J50% group; (**E**) J25% group. The direction and length of vectors indicate the weight of each variable in discriminating between groups.

Principal component analysis (PCA) confirmed factoriality (KMO = 0.716; Bartlett χ^2^(66) = 506.19, *p* < 0.001) and retained two components explaining 70.1% of the variance (PC1 = 54.5%, PC2 = 15.6%). After oblimin rotation, PC1 captured a nitro-oxidative/inflammatory axis with high loadings for IL-18, 3-NT, NF-κB, 8-OHdG, NO and MDA, inversely proportional to SH and TAC. PC2 reflected the oxidant/protein oxidative balance (TOS, OSI, AOPP ± IL-1β). Correlation circles (Figure 4) showed: INFL (A)—close correlation of oxidative/inflammatory markers on PC1 opposite to SH/TAC; diclofenac (B)—partial uncoupling, with TOS/OSI collinear on PC1 and NO/3-NT/8-OHdG/IL-18 opposite; J100 (C) pattern that has a behavior similar to the reference NSAID drug, with NO/IL-18/3-NT opposite TOS/SH; J50 (D), intermediate configuration (MDA/TAC/NO/IL-1β positive PC1; AOPP/SH/OSI negative; 3-NT/NF-κB oblique); J25 (E) produced the weakest reorganization. Therapeutic administration of the extracts shifted the biomarker interactions from the INFL profile towards partial restoration of the oxidant–antioxidant balance (Figure 4).

*Juniperus communis* extract modulates the oxidative inflammatory markers in a concentration-responsive manner. The highest concentration produced effects similar to diclofenac, while the extract with the lowest concentration produced the smallest impact from a statistical point of view.

**Figure 5:**
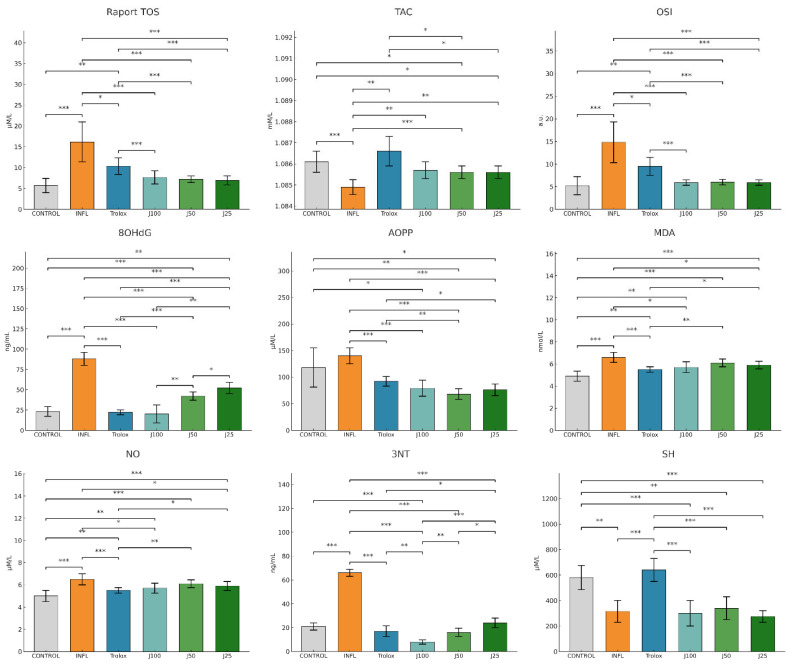
The prophylactic effect of *J. communis* extracts on oxidative stress parameters. * *p* < 0.05; ** *p* < 0.01; *** *p* < 0.001; Trolox—Trolox (50 mg/kg); J100—*J. communis* extract 100%; J50—*J. communis* extract 50%; J25—*J. communis* extract 25%; INFL—Inflammation induced by turpentine oil.

Following statistical analysis, it was found that the administration of turpentine oil triggered a statistically significant oxidative/nitrosative imbalance compared to CONTROL on all analyzed markers. Seven-day prophylactic administration of *J. communis* extract attenuated these changes in a concentration-dependent manner. J100 had a better cell protective effect. It prevented reduction in lipid and protein oxidation and nitrosative damage (MDA, AOPP, 3-NT, NO, 8-OHdG, OSI, TOS), while restoring TAC and SH to initial values (*p* < 0.01–0.001 compared to INFL) (Figure 5).

**Figure 6:**
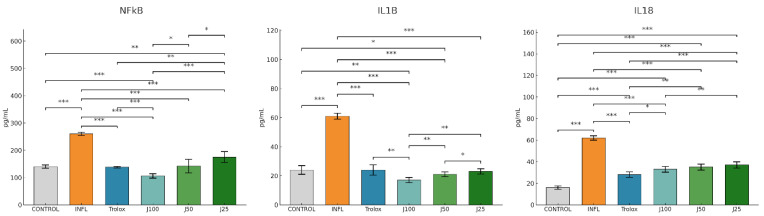
The prophylactic effect of *J. communis* extracts on oxidative stress parameters. * *p* < 0.05; ** *p* < 0.01; *** *p* < 0.001; NF-κB—Nuclear factor kappa B; IL-18—Interleukin 18; IL-1B—Interleukin 1 beta; Trolox—Trolox (50 mg/kg); J100—*J. communis* extract 100%; J50—*J. communis* extract 50%; J25—*J. communis* extract 25%; INFL—Inflammation induced by turpentine oil.

In agreement with the oxidative stress parameters, inflammatory markers (NF-κB, IL-1β, IL-18) were all increased in the INFL group compared to the CONTROL (*p* < 0.001). Pretreatment with *J. communis* improved the levels of these markers in a dose-dependent manner. J100 produced the greatest reduction (*p* < 0.01–0.001 compared to INFL), J50 remained significantly effective (*p* < 0.05–0.01), while J25 had a lesser impact on inflammatory markers (Figure 6).

Principal component analysis (PCA) was applied to explore the relationships between oxidative stress and inflammatory biomarkers and to assess the effects of prophylactic treatment. Since OSI~TOS/TAC, the combined correlation matrix showed close collinearity; to test the hypotheses, we confirmed the adequacy of sampling, KMO = 0.61, Bartlett χ^2^ (55) = 164.0, *p* < 0.001, supporting factoriality. Two principal components with eigenvalues > 1 were retained, explaining 71.7% of the variance (PC1 = 55.7%, PC2 = 16.0%).

**Figure 7:**
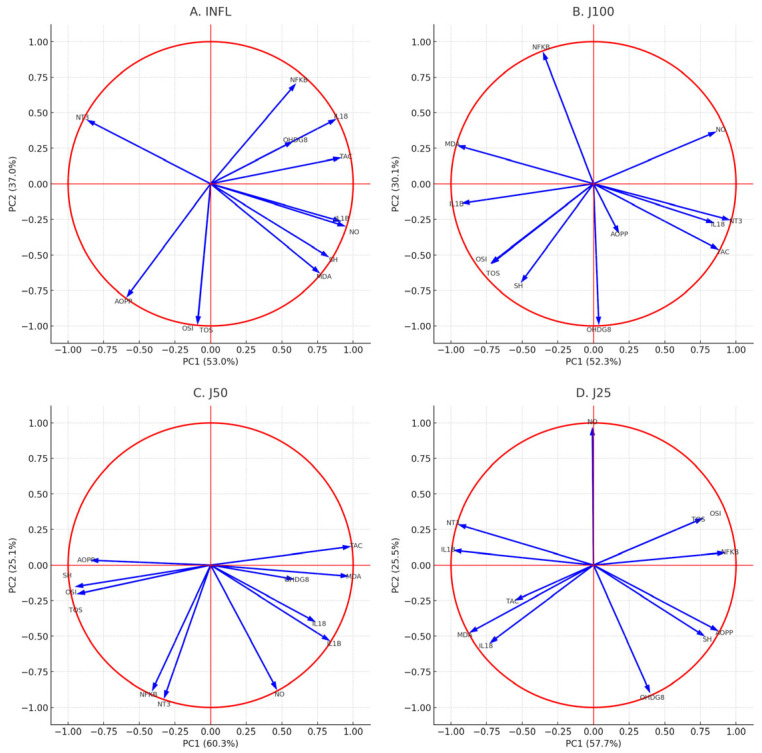
PCA correlation circles of oxidative stress and inflammatory markers in the prophylactic experimental setting. Vectors represent the contribution and correlation of each biomarker with the first two principal components (PC1 and PC2). (**A**) Inflammation group (INFL); (**B**) *J. communis* extract 100% group (J100); (**C***) J. communis* extract 50% group (J50); and (**D**) *J. communis* extract 25% group (J25). The orientation and length of the vectors indicate the degree of association of each variable with the principal components and their relative contribution to group discrimination.

Figure 7 illustrates how prophylactic administration of *J. communis* extract modulates the oxidative/inflammatory environment in a concentration-dependent manner: J100 shows the clearest shift towards a low antioxidant/inflammation profile, J50 is intermediate, and J25 produces a small effect.

## 3. Discussion

The results obtained in the present study demonstrate that *J. communis* exhibits statistically significant antioxidant and anti-inflammatory effects. These align with data reported in the literature [22].

In vitro tests confirmed the free radical-scavenging capacity of juniper extracts. For example, juniper berry extracts can scavenge DPPH radicals with IC50 values in the low μg/mL range, reflecting an efficacy comparable to standard antioxidants such as Trolox and quercetin [23]. This high radical-scavenging activity correlates with the rich polyphenol content of juniper.

Phytochemical analyses show that juniper is abundant in flavonoids (e.g., quercetin derivatives, catechins). In particular, the quercetin aglycone, identified in juniper extracts, is a flavonol with significant antioxidant action. The mechanism by which this is validated is hydroxyl groups that readily donate electrons to stabilize ROS [24]. There are data reporting positive correlations between juniper quercetin levels and DPPH scavenging or inhibition of lipid peroxidation [25]. Also, catechin-type polyphenols probably contribute to the radical-scavenging capacity, similar to the mechanism of epigallocatechin gallate in green tea. This catechin potently neutralizes free radicals by donating hydroxyl groups. In juniper, these phenols act synergistically with volatile terpenes and protect biomolecules from oxidative damage [26]. The combination of polyphenols in *J. communis* provides broad-spectrum antioxidant effects, as each class can quench reactive species and chelate pro-oxidant metals. This is evidenced by the ferric-reducing power of juniper extract and cellular antioxidant activity reported in our study and others [27]. Our phytochemical findings and in vitro results support this, suggesting that juniper needle extracts overlap in efficacy with conventional antioxidants in scavenging free radicals.

It should be emphasized that prophylactic and therapeutic effects represent distinct experimental paradigms, reflecting pre-exposure priming versus post-insult intervention, rather than interchangeable mechanisms. In our study, to highlight the effects in vivo, we administered *J. communis* extracts both therapeutically and prophylactically. Regardless of the purpose of therapeutic and prophylactic administration, they modulated the oxidative stress parameters towards the reference levels of the control groups. *J. communis* reduced TOS and increased TAC, leading to a lower OSI. This behavior of the extracts captures the direction of oxidative balance in vivo. The results of recent studies cited in the literature highlight that OSI demonstrates its utility for monitoring oxidative stress and the response to antioxidant interventions in clinical and experimental settings. The levels of these parameters obtained with the administration of *J. communis* are congruent with data reported by researchers in the academic literature and demonstrate that they had the ability to restore redox homeostasis [28].

In the case of prophylactic administration, the extracts probably primed the endogenous defense and/or buffered the initial burst of ROS/RNS. Measuring the parameters in the arm where the treatment was administered for therapeutic purposes facilitated the elimination of existing oxidants and accelerated the resolution of the oxidative phase of acute inflammation. The differences between the results obtained from the two protocols were modest, suggesting that *J. communis* can act both preventively and therapeutically. A similar dual approach has been reported for another plant rich in polyphenols/alkaloids, namely the bark of *Phellodendron amurense*. Within the administration schemes, both prophylactic and therapeutic schemes reduced the oxidative load and inflammatory mediators investigated by the authors: NO, 3-NT, and NF-κB. It was also observed that the benefits of administering the extracts are generally comparable between the two types of administration [29].

In the same dose-dependent manner, administration of *J. communis* needle extract reduced 8-OHdG in our study, indicating attenuation of oxidative DNA damage. This observation is in agreement with its validation as a sensitive in vivo biomarker of DNA oxidation, which decreases in response to effective antioxidant interventions. The academic world has reported a series of beneficial results obtained by administration of plant extracts. Green tea polyphenols reduced hepatic 8-OHdG levels while activating Nrf2 defense in aged mice with D-galactose [30]. Another experiment performed by administration of aqueous extract of Fructus Ligustri Lucidi reduced 8-OHdG in ovariectomized rat tissues [31]. Another conclusive example is the administration of pomegranate juice, which reduced retinal 8-OHdG levels in diabetic rats [32]. All these results support the observation that antioxidant extracts derived from plants, similar in phytochemistry to *J. communis*, can reduce 8-OHdG levels in vivo, strengthening our interpretation that the observed decline reflects real protection at the DNA level.

The decrease in AOPP obtained in our study indicates attenuation of protein oxidative damage and is consistent with in vivo evidence from botanical interventions. In an experimental model of acute inflammation, ethanolic extracts of *Artemisia dracunculus*/*abrotanum* reduced systemic oxidative stress that included both NO and AOPP [33]. In an experimental model of STZ-induced diabetes, oral *Colocasia affinis* leaf extract significantly reduced both NO and AOPPs, the authors concluded, attenuating nitro-oxidative protein damage [34]. All this evidence, supported by in vivo tests of various pathologies, supports our interpretation that the decrease in AOPPs observed during *J. communis* administration reflects a real attenuation of oxidative damage at the protein level in vivo.

The decrease in MDA in our experimental rats indicates reduced membrane lipid peroxidation, a pattern widely reproduced by in vivo phytotherapy administration. For example, defatted walnut kernel extracts reduced serum and tissue MDA in aged mice with D-galactose while stimulating endogenous antioxidant activities [35]. Another eloquent example in which flavonoid-rich extracts, such as our *J. communis* needle extract, are administered is *Sambucus nigra* extract. It reduced renal lipid peroxidation in a gentamicin nephrotoxicity model [36]. Together, these data reinforce the fact that polyphenol-rich plant extracts have a favorable impact on the MDA parameter in vivo tests. Therefore, the observations made by our results are in line with the results of the international academic community.

In vivo results also highlight the reduction in NO, derived largely from iNOS, but also the decrease of 3-NT, a stable signature of peroxynitrite attack. These results mirror observations made in several experiments administering phytotherapeutics. Green tea polyphenols and epigallocatechin gallate reduced colonic nitrotyrosine immunoreactivity and inflammatory indices in IL-2-KO mice with colitis [37]. A bark extract of *P. amurense* reduced NO, 3-NT and NF-κB in vivo, underlining the simultaneous control of nitrosative stress and inflammatory signaling [29]. Additional models show that botanical polyphenols reduce NO and peroxynitrite footprints, supporting our interpretation that reduction in NO availability and downstream 3-NT accompanies the anti-inflammatory action of plant extracts in vivo.

In our experimental model, administration of *J. communis* extract did not significantly restore serum total thiol (SH) levels compared with the inflammation group, yielding values comparable to those observed with diclofenac treatment. In contrast, several botanical extracts have been reported to increase thiol levels in vivo under different pathological contexts. For instance, in acetaminophen hepatotoxicity, *Stachys pilifera* extract increased total hepatic thiols and reduced protein carbonyls [38]. In an allergy-inflammation model, *Ocimum basilicum* (and rosmarinic acid) increased serum thiols while reducing NO and MDA. *Zataria multiflora* extract increased thiol levels in an Adriamycin cardiotoxicity model [39]. Taken together, our findings suggest that *J. communis* extract differs from classical thiol-restoring antioxidants, acting instead by limiting inflammation-driven oxidative stress and preserving thiol homeostasis indirectly, rather than by increasing thiol availability per se.

Administration of *J. communis* needle extract decreased NF-κB activity along with IL-1β and IL-18. Biologically, NF-κB primes the components of the inflammasome, and the NLRP3-caspase-1 complex cleaves pro-IL-1β and pro-IL-18 to their mature active forms. As a consequence, synchronous attenuation of NF-κB/IL-1β/IL-18 is expected from a mechanism of action perspective when redox signals and upstream pattern recognition are restricted. Our model aligns with anti-inflammatory data focused on administration in human dermal fibroblasts [40] and macrophage systems and abundant monoterpene drivers in *J. communis* (e.g., α-pinene, terpinen-4-ol) that negatively regulate NF-κB production and attenuate IL-1β/IL-18 production and attenuate porcine intestinal IL-1β/IL-18 production [41].

Principal component analysis (PCA) of our biochemical data suggested an interconnected oxidative inflammatory mechanism under *J. communis* treatment. The clustering of oxidative stress markers with inflammatory indices in the PCA space of control animals supports the idea that these pathways are closely linked, an observation consistent with other multivariate analyses of inflammation, as shown in Figure 4 and Figure 7. In the groups in which experimental animals received ethanolic *J. communis* extracts, this cluster shifted towards the profile of healthy controls, indicating a concordant normalization of both oxidative and inflammatory variables. In essence, *J. communis* extract appears to break the vicious circle of positive feedback between ROS and inflammation, shifting and homeostatic parameters. Interventions that primarily target either inflammation (e.g., NSAIDs) or oxidative stress (e.g., antioxidant agents such as vitamin E analogs) may not fully normalize both arms of the interconnected redox-inflammatory response. In contrast, dual modulation, as observed with juniper extract administration, may better disrupt this reciprocal amplification. This dual pattern is conceptually consistent with other phytochemicals reported to modulate the redox-inflammatory link, such as curcumin and green tea catechins, which can simultaneously reduce reactive species and dampen pro-inflammatory signaling (e.g., NF-κB/iNOS) [42,43]. the phytochemical profiling performed in the present study focused on phenolic constituents of the ethanolic *J. communis* needle extract and identified quercetin derivatives, taxifolin and flavan-3-ols as major components. Terpenoids were not quantified in this extract; therefore, mechanistic interpretations are restricted to the polyphenolic fraction, and no compositional comparison with other botanicals is made. This coordinated mechanism likely underlies the effective attenuation of oxidative stress and inflammation observed in our study.

Although the present study provides evidence that an ethanol-based *J. communis* needle extract can modulate oxidative/nitrosative stress and inflammatory endpoints in an acute turpentine oil-induced inflammation model, several limitations should be acknowledged. One of these is that we used an acute inflammation model and a single type of extract, namely ethanol-based extracts. Models of chronic pathologies (e.g., arthritis/colitis) and fractionation would clarify durability and tissue specificity. Another aspect is that, although the range of parameters was diversified, we did not directly measure the upstream enzymatic systems, iNOS/COX-2. Another point is the variability of the phytochemical batch and the season of harvesting conifer needles; the method chosen for extraction may alter the impact of TOS/TAC/OSI and cytokine modulation. Finally, we did not perform pharmacokinetic or toxicological studies.

Looking ahead, there are several avenues opened by this research. Testing *J. communis* extracts in models of chronic inflammation would clarify whether its dual antioxidant/anti-inflammatory effects can prevent progression to chronic pathology. On the other hand, long-term and dose-ranging studies are needed to establish the optimal therapeutic window and ensure safety. Animal administration studies (weeks to months) will shed light on whether *J. communis* has any chronic efficacy and whether toxicity occurs with prolonged use. Juniper extract is complex. Isolation of more active compounds could lead to more potent derivatives or help standardize extract preparations. The final step could be translating these findings into clinical use, but this will require rigorous studies in human subjects. Overall, these findings should be viewed as a proof-of-concept supporting further investigation rather than direct clinical translation.

## 4. Materials and Methods

### 4.1. Chemicals

Folin–Ciocâlteu reagent, sodium carbonate, sodium acetate, aluminum chloride, methanol, 2,2-diphenyl-1-picrylhydrazyl (DPPH), Griess-Ilosvay nitrite reagent, sodium nitroprusside, phosphate-buffered saline, *N*-(1-Naphthyl) ethylenediamine dihydrochloric acid (NEDD), sulphanilic acid, hydrogen peroxide (H_2_O_2_), 2,4,6-tri(2-pyridyl)-1,3,5-triazine (TPTZ), acetate buffer, ferric chloride, xylenol orange, ortho-dianisidine dihydrochloride (3-3′-dimethoxybenzidine), thiobarbituric acid, ethylenediaminetetraacetic acid, sodium dodecyl sulfate, butylated hydroxytoluene, thiobarbituric acid, 1,1,3,3-tetrahydroxypropane, Vanadium III chloride (VCl3), 5,5′-di-thio-bis 2-nitrobenzoic acid (DTNB) and Trolox (6-hydroxy-2,5,7,8-tetramethylchroman-2-carboxylic acid) were purchased from Merck (Darmstadt, Germany) and Sigma-Aldrich (Munich, Germany). Acetonitrile, of HPLC purity, was purchased from Merck (Darmstadt, Germany), and ultrapure water was purified with the Direct-Q UV system from Millipore (Burlington, MA, USA). Standard gallic acid (>99% HPLC), catechin (>99% HPLC), and rutin (>94% HPLC) were purchased from Sigma (St. Louis, MO, USA). The protocol adhered to the manufacturer’s guidelines, and absorbance was recorded at 450 nm. Results were reported in nanomoles per millilite ELISA kits for rat 3-NT (EU2560, FineTest, Wuhan, China), rat 8-OHdG (E-EL-0028, E-EL-R0371, Elabscience Innovation Bionovation Inc., Houston, TX, USA), rat NF-κB p65 (ER1187, FineTest, Wuhan, China), rat IL1β (ER1094, FineTest, Wuhan, China), rat IL-18 (ER1094, FineTest, Wuhan, China), and rat caspase 1 (E-EL-R0371, Elabscience Innovation Bionovation Inc., Houston, TX, USA) were used.

### 4.2. Extract Preparation

Fresh needles of *J. communis* L. were sourced from the “Alexandru Borza” Botanical Garden, Cluj-Napoca, Romania (46°45′36″ N and 23°35′13″ E). Taxonomic identity was confirmed by a qualified botanist, and a voucher specimen (voucher Nr. 674529/2023) was deposited in the Herbarium of the same institution. Immediately after harvesting, needles were separated, trimmed into 0.5–1.0 cm segments, and processed in the Mycology Laboratory of Babeș-Bolyai University (Cluj-Napoca, Romania). Extraction was performed with 70% ethanol (Merck, Bucharest, Romania) using a repercolation protocol adapted from the Squibb method. Briefly, plant material was loaded into three percolators as follows: 150 g in the first one, 90 g in the second one, and 60 g in the third one. Then plant material was soaked with 150 mL of 70% ethanol, and after two days, the three percolated fractions (60 mL, 90 mL, and 120 mL) were collected and mixed. The resulting final extract had a concentration of 1:1 g/mL (*w*:*v*) in 30% ethanol. The final extract was stored at 4 °C until further analysis. [44].

### 4.3. Determination of Total Phenolic (TPC) and Total Flavonoid Content (TFC)

TPC of the *J. communis* ethanolic extract was quantified by the Folin–Ciocâlteu assay using gallic acid as the external standard. In brief, 2.00 mL of extract was combined with 1.00 mL of Folin–Ciocâlteu reagent and 10.0 mL of deionized water; the mixture was then brought to 25.0 mL with sodium carbonate solution (290 g/L). After a 30 min incubation at room temperature in the dark, absorbance was recorded at 760 nm on a UV-Vis spectrophotometer (JASCO V-530; Jasco, Tokyo, Japan). Results were calculated from a gallic acid calibration curve (R^2^ = 0.999) and expressed as milligrams of gallic acid equivalents per gram of extract dry weight (mg GAE/g d.w.) [45].

TFC was determined by the aluminum chloride colorimetric method. Appropriate aliquots of the extract were reacted with aluminum chloride and sodium acetate solutions, diluted to volume, and the absorbance was measured at 430 nm. Quantification used a quercetin standard curve (R^2^ = 0.999), with data expressed as milligrams of quercetin equivalents per gram of extract dry weight (mg QE/g d.w.). Each determination was carried out in triplicate [46].

### 4.4. HPLC-DAD-ESI MS

An Agilent 1200 HPLC system from Agilent Technologies (Santa Clara, CA, USA), with a quaternary pump, solvent degasser, autosampler, UV-Vis diode-array detector (DAD), and single-quadrupole mass spectrometer (Agilent 6110), was utilized for compound analysis. Separation was conducted using a Kinetex XB C18 column (4.6 × 150 mm, 5 μm; Phenomenex, Torrance, CA, USA). The mobile phase comprised (A) water with 0.1% acetic acid and (B) acetonitrile with 0.1% acetic acid, administered at a flow rate of 0.5 mL/min and a column temperature of 25 °C, utilizing the following gradient (in % B): 0 min, 5%; 0–2 min, 5%; 2–18 min, 5–40%; 18–20 min, 40–90%; 20–24 min, 90%; 24–25 min, 90–5%; and 25–30 min, 5%. UV spectra were obtained in the range of 200–600 nm, while chromatograms were observed at 280 and 340 nm [47].

Mass spectrometry (MS) was conducted in full-scan positive electrospray ionization (ESI) mode with the following parameters: capillary voltage at 3000 V, source temperature at 350 °C, nitrogen flow at 7 L/min, and scan range from *m*/*z* 120 to 1200. Data acquisition and processing were performed utilizing Agilent ChemStation software (Rev B.04.02 SP1). Compound identification relied on retention time, UV-Vis spectra, mass spectra, and pertinent literature data [48].

Three standards were used for quantification: gallic acid (R^2^ = 0.99; LOD = 0.35 μg/mL; LOQ = 1.05 μg/mL) for hydroxybenzoic acids, catechin (R^2^ = 0.99; LOD = 0.18 μg/mL; LOQ = 0.72 μg/mL) for flavanols, and rutin (R^2^ = 0.99; LOD = 0.21 μg/mL; LOQ = 0.84 μg/mL) for flavonols.

### 4.5. In Vitro Testing of Total Antioxidant Capacity

The antioxidant activity was assessed by various in vitro tests: 2,2-diphenyl-1-picrylhydrazyl (DPPH) radical-scavenging, ferric-reducing antioxidant power (FRAP), nitric oxide (NO) scavenging, and hydrogen peroxide (H_2_O_2_) scavenging assays. All in vitro tests were conducted in triplicate.

#### 4.5.1. DPPH

The DPPH Radical-Scavenging Assay consisted of combining equal amounts of the sample solution with 0.1 mg/mL DPPH in methanol, followed by a 30 min incubation in the dark at room temperature. The absorbance at 517 nm was quantified. The antioxidant capacity was measured as IC50 values (μg/mL) and converted to TE (μg TE/mL) via a calibration curve. According to the TE values, antioxidant potential was categorized as very good (<50 μg TE/mL), good (50–100 μg TE/mL), weak (100–200 μg TE/mL), or negligible (>200 μg TE/mL) [49].

#### 4.5.2. FRAP

The FRAP assay evaluated antioxidant activity by measuring the reduction of Fe^3+^ to Fe^2+^ utilizing TPTZ. A reagent mixture including acetate buffer, TPTZ, and ferric chloride in a ratio of 10:1:1 (*v*/*v*/*v*) was incubated for 30 min, and absorbance was measured at 593 nm. Results were quantified as milligrams of trolox equivalents (TE) per milliliter of ethanolic plant extract (mg TE/mL) [50].

#### 4.5.3. Nitric Oxide (NO)

Nitric oxide scavenging activity was assessed using the Griess reagent method [51]. Ethanolic extract of *J. communis* was incubated with sodium nitroprusside (SNP) and phosphate-buffered saline (PBS) for 2.5 h. The resultant mixture was further reacted with sulphanilic acid and *N*-(1-Naphthyl) ethylenediamine dihydrochloride (NEDD). Following a concluding 30 min incubation, absorbance was assessed at 546 nm, and the data were articulated as IC50 values. Results were presented as μmol Quercetin equivalents per milliliter of ethanolic plant extract (μmol QE/mL).

#### 4.5.4. Hydrogen Peroxide (H_2_O_2_) Scavenging Activity

The capacity of *J. communis* ethanol extracts to scavenge hydrogen peroxide (H_2_O_2_) was determined as previously outlined. The extracts were combined with an H_2_O_2_ solution, and absorbance was recorded at 230 nm using a phosphate buffer blank after 10 min. The proportion of H_2_O_2_ scavenging was estimated using the method % scavenged H_2_O_2_ = (A control − A sample)/A control × 100. The data were subsequently presented as IC50 in micrograms of Trolox equivalent per milliliter (μg TE/mL) [52].

### 4.6. Experimental Design In Vivo

Male Wistar rats (200–250 g) were obtained from the Center for the Breeding and Use of Laboratory Animals, “Iuliu Hațieganu” University of Medicine and Pharmacy, Cluj-Napoca, Romania. Animals were housed in standard polycarbonate cages under controlled conditions (22 ± 2 °C; 50 ± 10% relative humidity; 12 h light/12 h dark photoperiod) with ad libitum access to food and water. Bedding was changed regularly, and all animals were acclimatized for at least one week prior to experimentation.

All procedures conformed to national regulations and international guidelines for the care and use of laboratory animals. The protocol was reviewed and approved by the Institutional Ethics Committee of “Iuliu Hațieganu” University of Medicine and Pharmacy (No. 373/07.2023). Throughout the study, animals were monitored daily, and predefined humane endpoints were applied. All efforts were made to minimize suffering, reduce the number of animals used, and refine procedures. Randomization was used for group allocation.

Two complementary protocols were employed to evaluate the therapeutic and prophylactic effects of the *J. communis* needle extract, using constant extract group labels across both arms (group J100, corresponding to undiluted extract treatment; group J50, corresponding to 50% concentration extract treatment; and group J25, corresponding to 25% concentration extract treatment). Treatments were performed by oral gavage (1 mL/rat/day). Animals were randomized (n = 5/group) and handled under identical housing and monitoring conditions. Acute inflammation was induced with turpentine oil (6 mL/kg, intramuscular, right hind paw), whereas non-inflamed controls were not challenged.

#### 4.6.1. Protocol for Evaluating Therapeutic Effect

Acute inflammation was induced on Day 1 in all groups except Control. Beginning on Day 2 and continuing through Day 8, daily treatments were administered as follows: DICLO received diclofenac (10 mg/kg/day), Troloxreceived Trolox (50 mg/kg/day), and the extract groups received *J. communis* at the designated concentrations (J100, J50, J25); Controland inflammation (INFL) received tap water.

#### 4.6.2. Protocol for Evaluating Prophylactic Effect

Daily oral dosing was given on days 1–7 to a non-inflamed Control (tap water), an INFL control (tap water), a Troloxcomparator (50 mg/kg/day), and the extract groups J100, J50, and J25. On Day 8, all prophylaxis groups except the non-inflamed CONTROL were challenged with turpentine oil as above.

For both arms, terminal procedures were performed on Day 9 under ketamine (60 mg/kg) and xylazine (15 mg/kg) anesthesia (intraperitoneal), followed by retro-orbital blood collection, serum separation, and storage at −20 °C.

### 4.7. Oxidative Stress Marker Assessment

#### 4.7.1. Total Oxidative Status (TOS)

TOS was assessed with a colorimetric technique that quantifies oxidants by converting ferrous ions (Fe^2+^) to ferric ions (Fe^3+^) in an acidic environment, identified through a xylenol orange reaction. An automated analyzer, calibrated with hydrogen peroxide, was utilized, and results were reported in μM H_2_O_2_ equivalents per liter [53].

#### 4.7.2. Total Antioxidant Capacity (TAC)

TAC is indicative of overall antioxidant defenses and was assessed using a colorimetric technique. The assay evaluated the ability to neutralize dianisidyl radicals produced by the oxidation of ortho-dianisidine. A typical solution of Fe^2+^-o-dianisidine engages in a Fenton reaction with hydrogen peroxide, yielding hydroxyl radicals. The antioxidants in the sample impede the oxidation of o-dianisidine, hence diminishing color development. The color intensity was quantified spectrophotometrically, and the assay was calibrated with Trolox. TAC values were represented as mM Trolox equivalents (TE) per liter [54].

#### 4.7.3. The Oxidative Stress Index (OSI)

OSI, which indicates the equilibrium between oxidants and antioxidants, was calculated by dividing the total oxidant status (TOS) value (mM H_2_O_2_ equivalents/L) by the total antioxidant capacity (TAC) value (mM TE/L) [55].

#### 4.7.4. Malondialdehyde (MDA)

MDA, a marker of lipid peroxidation, was measured utilizing a thiobarbituric acid test. One hundred fifty microliters of serum were combined with one hundred twenty-five microliters of 10% trichloroacetic acid, one hundred twenty-five microliters of 5 mM EDTA, one hundred twenty-five microliters of 8% sodium dodecyl sulfate, and ten microliters of 0.5% butylated hydroxytoluene. Following incubation and centrifugation, the supernatant was treated with thiobarbituric acid (500 μL of 0.6%) at 95 °C. Absorbance was quantified at 532 nm, and MDA concentrations were ascertained utilizing a 1,1,3,3-tetrahydroxypropane standard curve (0.3–10 nM/mL), with data reported as nM/mL serum [56,57].

#### 4.7.5. 8-Hydroxydeoxyguanosine (8-OHdG)

8-OHdG levels, a biomarker indicative of oxidative DNA damage, were quantified utilizing an ELISA kit Rat 8-OHdG kit (catalog No: ER1487, Wuhan, China) in accordance with the manufacturer’s guidelines. Serum samples and standards were introduced into wells pre-coated with an 8-OHdG conjugate, subsequently incubated with a particular antibody. Absorbance was measured at 450 nm, and concentrations were reported in ng/m.

#### 4.7.6. Advanced Oxidation Protein Products (AOPP)

Serum advanced oxidation protein products (AOPP) were quantified using a method modified from Witko-Sarsat et al. [38]. Serum samples were diluted in phosphate-buffered saline and reacted with glacial acetic acid and potassium iodide. Absorbance was quantified at 340 nm. AOPP concentrations were quantified as µM chloramine-T equivalents per liter.

#### 4.7.7. Total Thiols (SH)

SH concentrations in serum were measured utilizing Ellman’s reagent [58]. The reagent interacts with thiol groups, and the absorbance of the resultant supernatant was quantified spectrophotometrically at 412 nm. SH concentrations were quantified as mM of reduced glutathione (GSH) per mL [58].

#### 4.7.8. 3-Nitrotyrosine (3-NT)

Concentrations of 3-NT, an indicator of protein nitration, were assessed utilizing a competitive ELISA kit (catalog No: EU2560, FineTest, Wuhan, China). The protocol adhered to the manufacturer’s guidelines, and absorbance was recorded at 450 nm. Results were reported in nanomoles per milliliter [59].

### 4.8. Assessment of Inflammatory Markers

A sandwich ELISA (Rat NF-κB p65 kit (catalog No: ER1187, FineTest, Wuhan, China)) was employed to quantify the activity of the NF-κB p65 subunit, following the manufacturer’s guidelines. The samples designated for analysis were positioned in wells containing a specific antibody, subsequently identified using a secondary antibody, followed by detection using a biotinylated secondary antibody and HRP-conjugated streptavidin. The data were expressed in pg/mL following the absorbance measurement at 450 nm. High-sensitivity kits were employed for the two interleukins. IL1β concentrations (Rat IL-1b ELISA kit, catalog No: ER1094, FineTest, Wuhan, China) and IL-18 (Rat IL-1b ELISA kit, catalog No: ER1094, FineTest, Wuhan, China) were quantified using a sensitivity ELISA kit, adhering to the manufacturer’s guidelines. Absorbance was measured at 450 nm, and concentrations were reported in pg/mL.

### 4.9. Statistical Analysis

Data analysis was carried out using SPSS v26.0 (SPSS Inc., Chicago, IL, USA) and R v.5.1 (R Foundation for Statistical Computing, Vienna, Austria). For variables conforming to normal distributions, results are reported as mean ± standard deviation (SD). Group differences were assessed by one-way ANOVA followed by Holm–Bonferroni-adjusted post hoc pairwise comparisons. Associations between biomarkers were examined with Pearson correlation. Statistical significance was defined as *p* < 0.05. For multivariate exploration, Principal Component Analysis (PCA) was performed in order to find the correlation between measured parameters and to check their variability in the rat groups according to different concentrations in plant extract administration. A positive correlation between parameters is suggested by an angle < 90° between the two vectors, and a negative correlation between parameters is suggested by an angle > 90° and close to 180° between the two vectors. The variability of these parameters was explained by the comparisons of the first principal component (PC1) and the second one (PC2), as shown in the score plots. Data suitability for factor analysis was verified with Bartlett’s test of sphericity, and sampling adequacy was evaluated using the Kaiser–Meyer–Olkin (KMO) criterion [33].

## 5. Conclusions

This study demonstrates that an ethanol-based *Juniperus communis* needle extract is able to modulate the redox-inflammatory response in an experimental model of acute inflammation. The observed effects indicate that targeting oxidative stress and inflammatory signaling simultaneously may represent an effective strategy in limiting acute inflammatory damage. The concentration-dependent responses observed in vivo, together with the multivariate integration of biochemical markers, suggest that the extract influences interconnected oxidative and inflammatory pathways rather than isolated endpoints. Conclusions of the present work are restricted to an acute experimental setting and to an ethanol-based extract characterized by its phenolic composition. The lack of chronic models, fractionation, pharmacokinetic, and toxicological evaluation represents a limitation that should be addressed in future studies. Further research should focus on long-term administration, safety profiling, and extract standardization, as well as on identifying the specific phenolic constituents responsible for the observed biological effects. Such studies are required before any translational or clinical relevance can be inferred.

## Figures and Tables

**Table 1:** HPLC–DAD–ESI–MS profile of phenolic compounds in *J. communis* ethanolic extract.

Peak No	Rt (min)	UV λmax (nm)	[M+H]^+^ (*m*/*z*)	Compound	Concentration (μg/mL)
1	3.12	275	155	2,3-Dihydroxybenzoic acid ^1^	49.38 ± 0.88
2	9.55	280	307	Gallocatechin ^2^	65.93 ± 2.37
3	10.48	275	155	2,4-Dihydroxybenzoic acid ^1^	7.97 ± 0.32
4	11.38	280	579	Procyanidin dimer B3 ^2^	41.66 ± 1.49
5	11.94	280	579	Procyanidin dimer B1 ^2^	54.66 ± 0.74
6	12.25	280	291	Catechin ^2^	136.51 ± 4.46
7	12.83	280	579	Procyanidin dimer B2 ^2^	60.25 ± 6.97
8	13.51	280	291	Epicatechin ^2^	58.21 ± 0.09
9	14.58	358, 255	521, 317	Isorhamnetin-acetyl-glucoside ^3^	23.99 ± 0.08
10	14.96	350, 260	579, 287	Kaempferol-rhamnosyl-rhamnoside ^3^	48.87 ± 0.18
11	15.61	350, 250	465, 319	Myricetin-rhamnoside ^3^	187.09 ± 2.60
12	15.86	360, 255	465, 303	Quercetin-glucoside ^3^	63.70 ± 0.47
13	17.12	360, 255	449, 303	Quercetin-rhamnoside ^3^	262.12 ± 3.86
14	23.09	350, 260	287	Kaempferol ^3^	116.87 ± 3.41
15	23.44	360, 270	305	Taxifolin ^3^	197.91 ± 2.15

^1^—hydroxybenzoic acid compounds, ^2^—flavanol compounds, ^3^—flavonol compounds; results are presented as mean ± SD.

**Table 2:** Antioxidant and anti-inflammatory activity in vitro of *Juniperus communis* ethanolic extract.

Sample/Standard	DPPH (µg TE/mL)	H_2_O_2_ (µg TE/mL)	FRAP (mg TE/mL)	NO (µg QE/mL)
Extract	80.41 ± 8.34	128.03 ± 15.03	112.87 ± 28.82	64.60 ± 5.56
Trolox	11.20 ± 0.45	24.23 ± 1.53	19.97 ± 1.10	-
Quercetin	-	-	-	20.58 ± 2.68
*p* value	<0.01	<0.01	0.030	<0.01

TE = Trolox equivalents; QE = quercetin equivalents; DPPH = 2,2-diphenyl-1-picrylhydrazyl radical scavenging; H_2_O_2_ = hydrogen peroxide scavenging activity; FRAP = ferric-reducing antioxidant power; NO = nitric oxide scavenging activity.

## Data Availability

Data are available only for reviewers until the first author defends his Ph.D. thesis.
